# Hepaticojejunostomy with gastric access loop versus conventional hepaticojejunostomy: a randomized trial

**DOI:** 10.1007/s13304-023-01604-6

**Published:** 2023-08-09

**Authors:** Mohamad Raafat, Morsy M. Morsy, Salah I. Mohamed, Mostafa A. Hamad, Mostafa M. Sayed

**Affiliations:** Department of General Surgery, Faculty of Medicine, Assiut University, 10 Building W, Assiut University Campus, Asyut, 71515 Egypt

**Keywords:** Roux-en-Y hepaticojejunostomy, Hepaticojejunostomy anastomotic stricture, Gastric access loop

## Abstract

Roux-en-Y hepaticojejunostomy (RYHJ) with the provision of “gastric access loop” was developed to shorten the distance traveled by the endoscope to reach hepaticojejunostomy (HJ) anastomotic site. The aim of our study was to assess modified RYHJ with gastric access loop (RYHJ-GA) and compare it with conventional RYHJ (RYHJ-C) regarding short- and long-term outcomes and, moreover, to evaluate the feasibility and results of future endoscopic access of the modified bilio-enteric anastomosis. Patients eligible for RYHJ between September 2017 and December 2019 were allocated randomly to receive either RYHJ-C or RYHJ-GA. Fifty-two patients were randomly assigned to RYHJ-C (*n* = 26) or RYHJ-GA (*n* = 26). Three cases in RYHJ-C and 4 cases in RYHJ- GA developed HJ anastomotic stricture (HJAS) (*P*=0.68). 3 cases of RYHJ-GA had successful endoscopic dilation and balloon sweeping of biliary mud (one case) or stones (2 cases). Revisional surgery was needed in 2 cases of RYHJ-C and 1 case in RYHJ-GA (*P*=0.68). Modified RYHJ with gastric access loop is comparable to the classic hepaticojejunostomy regarding complications. However, gastric access enables easy endoscopic access for the management of future HJAS. This modification should be considered in patients with a high risk of HJAS during long-term follow-up.

*The trial registration number (TRN) and date of registration*:ClinicalTrials.gov (NCT03252379), August 17, 2017.

## Introduction

Roux-en-Y hepaticojejunostomy (RYHJ) is the standard procedure used by most hepatobiliary surgeons for biliary reconstruction during surgical management of iatrogenic bile duct injury, benign biliary strictures, choledochal cysts and biliary tract tumors. Hepaticojejunostomy anastomotic stricture (HJAS) is the most serious long-term complication due to its devastating sequelae [1]. Unfortunately, the incidence of HJAS in experienced centers is significantly high ranging between 10 and 22% [2–7].

Although HJAS can be treated in selected cases by interventional radiology with percutaneous transhepatic balloon dilation, or endoscopically using a long enteroscope, the performance of a new biliary-enteric anastomosis is the most widely used therapeutic option [8–15]. However, revisional surgery is technically demanding, and associated with high morbidity and mortality rates [5, 6, 16].

Despite the high efficacy of endoscopic management of HJAS via either balloon dilatation or stenting, the endoscopic access to the anastomotic site is hampered by the long distance traveled by the endoscope in the jejunal loop to reach the bilio-enteric anastomosis. This can be overcome if a short “access loop” to bilio-enteric anastomotic site is available. The provision of gastric access loop adjunct to RYHJ may facilitate endoscopic intervention by allowing bile duct cannulation via a short gastro-jejunal pathway [17–22] using a standard gastroduodenoscope without the need for a sophisticated long enteroscope.

The aim of our study was to assess modified RYHJ with gastric access loop (RYHJ-GA) and compare it with conventional RYHJ (RYHJ-C) regarding short- and long-term outcomes and, moreover, to evaluate the feasibility and results of future endoscopic access of the modified bilio-enteric anastomosis.

## Patients and methods

This study is a prospective randomized trial comparing patients undergoing hepaticojejunostomy with or without gastric access loop. All patients who were eligible for RYHJ reconstruction at the General surgery department, Assiut University Hospitals between September 2017 and December 2019 have been recruited. Excluded from the study were patients with malignant diseases due to poor patient survival and difficulty to differentiate between anastomotic stricture and local anastomotic recurrence [23, 24], and also any case with markedly dilated CBD (> 20mm) due to extremely low risk for those patients to develop anastomotic stricture [25, 26]. The study protocol was approved by the Institutional Review Board of the Faculty of Medicine, Assuit University. The trial was registered at ClinicalTrials.gov (NCT03252379). An informed written consent was obtained from each patient before inclusion. The patients were allocated randomly to receive either RYHJ-C or RYHJ-GA by means of sealed envelopes. Block randomization was used to achieve a balance between study groups.

### Pre-operative preparation

For all patients, full medical history, clinical examination, routine laboratory investigations including liver function tests were performed. Imaging studies were also carried out in the form of abdominal ultrasonography (US), computerized tomography scan (CT) of the abdomen and magnetic resonance cholangiography (MRC) if indicated.

Endoscopic retrograde cholangiopancreatography (ERCP) was performed in most cases whether for diagnosis or therapeutic trial. All cases received low molecular weight heparin (Clexane^®^ 40 mg) 12 hrs before surgery as prophylaxis against deep venous thrombosis (DVT).

### Surgical technique

**Fig. 1:**
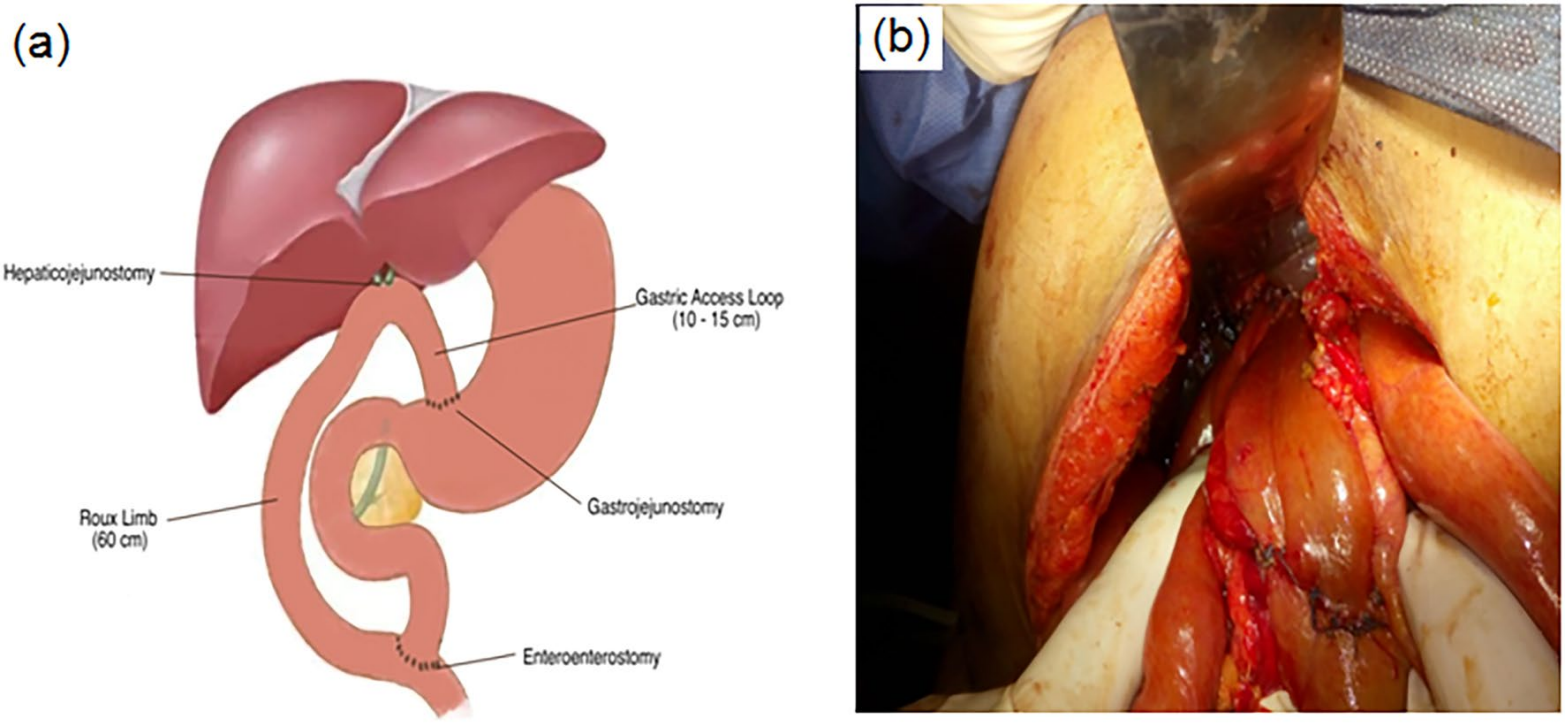
Hepaticojejunostomy with gastric access loop. **a** Illustrative diagram. **b** Operative view showing hepaticojejunostomy and gastrojejunostomy

Under general anesthesia, a right subcostal incision was performed and could be extended on demand upward to the xiphoid process and/or to the left subcostal area. Adhesiolysis of preformed adhesions if present and cholecystectomy were performed unless the gallbladder had already been removed. Cautious and thorough dissection was performed to reach the common bile duct (CBD) and common hepatic duct (CHD) and prepare a healthy proximal CHD for bilio-enteric anastomosis. Ductoplasy was performed when needed to provide single and wide anastomosis. For RYHJ-GA, a Roux jejunal loop of 70 cm length was prepared and passed retro colic to reach the porta hepatis. Then, the hepaticojejunostomy (HJ) was done via end-to-side anastomosis using interrupted sutures of polyglactin 4–0 size. The anastomosis was done 10–15 cm away from the free end of the Roux jejunum loop to allow anastomosis without tension to the stomach. The end of the Roux jejunal loop taken up for hepaticojejunostomy was not closed but was anastomosed to the anterior wall of the gastric antrum about 5cm proximal to the pyloric orifice (Fig. 1). For gastrojejunostomy (GJ), a gastrostomy incision was adapted to the diameter of jejunal end and the anastomosis was formed by continuous layer of polyglactin 3–0 size, reinforced by few interrupted sutures. Two intraperitoneal drains were placed in the hepatorenal pouch and pelvis before closing the abdomen. Nasogastric tube was left for decompression till the patient regained normal bowel movement.

### Post-operative follow-up

All patients were tested for liver function tests and abdominal ultrasonography was performed at the end of the 2nd post-operative week. Patients were reviewed 6 weeks after surgery, then after 3 months, and at 6-month intervals thereafter, or whenever they became symptomatic.

Patients of the RYHJ-GA group underwent a trial of endoscopic assessment after 3 months postoperatively.

If obstructive jaundice, biliary pain, cholangitis, or persistent elevation of liver function tests, subsequently developed during follow-up, an abdominal ultrasound followed by MRCP was then carried out for evaluation of HJAS development. Moreover, in the RYHJ-GA group, endoscopic assessment of the HJ anastomotic site was done by the use of upper endoscopy.

Primary outcome measures were the incidence of cholangitis, HJAS and need for revisional surgery in both groups, and the feasibility of endoscopic access in the RYHJ-GA group. Secondary outcome measures included: operative parameters (operative time and intraoperative blood loss), short-term morbidities (biliary leak, gastrojejunostomy leak, ileus and surgical site infection), length of hospital stay, and long-term morbidities including incidence of incisional hernia in both groups and development of biliary gastritis in RYHJ-GA group.

### Endoscopic technique

An end-view gastroduodenoscope was used in all cases. The endoscope was introduced through the esophagus to the stomach where the endoscopist assessed the amount of bile reflux in the stomach. A scale of 0 to 2 was used where 0 means no bile, 1 means a minimal amount of bile staining the gastric mucosa, and 2 means a large amount of bile needed to be sucked [17]. Endoscopic evidence of gastritis was assessed and if present, the grade of gastritis was assigned according to the endoscopy-based Kyoto classification score of gastritis [27]. Then, the gastric access loop was entered via the gastroenterostomy to reach the HJ site. When the HJ stoma was reached, we injected a diluted contrast into the cannulated bile ducts using an ERCP catheter to obtain cholangiography. Failure of endoscopic access was defined as failure to reach the HJ and perform cholangiography. For failed cases, the cause of failure was reported. If the endoscopist diagnosed stricture and/or hepatolithiasis, a therapeutic trial was performed with balloon dilatation of stricture and/or retrieval of stones.

### Statistical analysis

Sample size calculation was based on the incidence of cholangitis as the primary outcome. The incidence of cholangitis in RYHJ-C was supposed to be 7.7% according to the results of a previous study [28]. We assumed that a direct connection between the stomach and bile duct in RYHJ-GA would increase the risk of cholangitis by 30 percent. Using G power 3.1.9 software, it was estimated that a sample size of 24 patients in each group would be required to achieve α error of 0.05 with a power of 80%.

Prospectively collected data were expressed as numbers and percentages for qualitative variables (which were compared by the Chi-square test) or as mean ± standard deviation for quantitative variables (which were tested by Mann– Whitney *U* test). For all statistical tests done, the threshold of significance was fixed at a 5% level (2-tailed unless otherwise specified). All analyses were performed using the IBM SPSS Statistics ver. 24.0 (NY, USA: IBM Corp.).

## Results

**Fig. 2:**
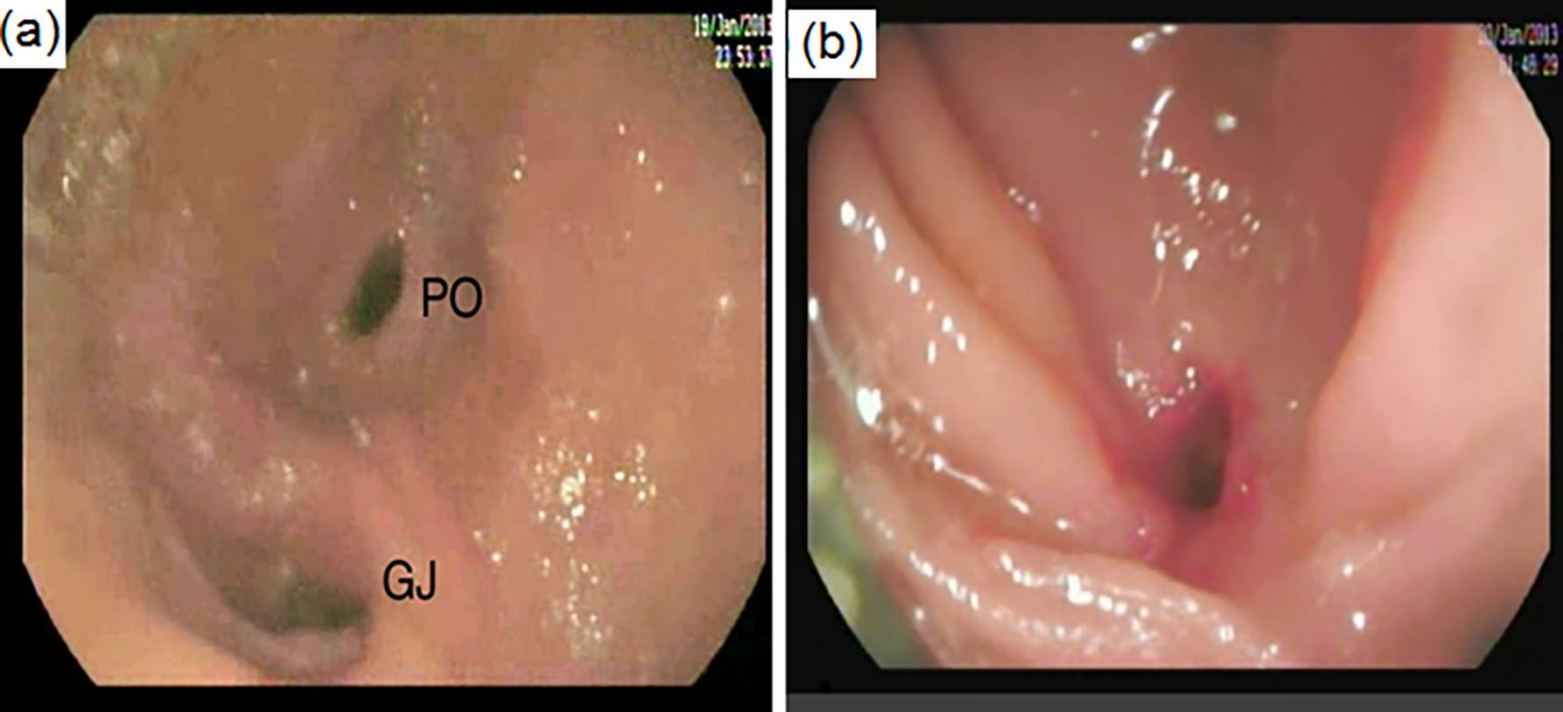
Endoscopic view in a patient who underwent biliary reconstruction with gastric access loop. **a** Gastrojejunostomy (GJ) and pyloric orifice (PO). **b** Hepaticojejunostomy

Between September 2017 and December 2019, a total of 70 patients were assessed for eligibility. Eighteen patients were excluded from the study (11 patients had malignant disease, 5 had dilated CBD > 20mm and 2 patients refused the study). Fifty-two patients were randomly assigned to RYHJ-C (*n* = 26) or RYHJ-GA (*n* = 26). The consort flow diagram is presented in Fig. 2.

### Patients’ demographic data

Majority of our patients were females (73.1%) and most of them underwent RYHJ due to iatrogenic bile duct injury (76.9%) with Type E2 injury represented the dominant class (47.5%). Other indications of surgery included benign biliary stricture (13.5%) and choledochal cyst type 1 (9.6%). There were no significant differences between the two groups regarding mean age, gender distribution, ASA class, indications for biliary shunt, mean CBD diameter, results of preoperative liver function tests, and ERCP performance as shown in Table 1.

**Table 1:** Clinico-epidemiologic data of the patients

		RYHJ-C (*n* = 26)	RYHJ-GA (*n *= 26)	*P* value
Age (years)	Mean ± SD	41.6 ± 6.4	43.1 ± 8.2	0.47
Gender	Male	8 (30.7%)	6 (23.1%)	0.53
	Female	18 (69.2%)	20 (76.9%)	
ASA class				0.68
	Class I	20	19	
	Class II	4	6	
	Class III	2	1	
BMI	Mean ± SD	28.2 ± 3.1	26.7 ± 2.8	0.073
Diagnosis				0.80
	CBD injury	19	21	
	E2 E3 E4	8 9 2	11 7 3	
	Choledochal cyst type1	3	2	
	Benign biliary stricture	4	3	
CBD diameter (mm)	Mean ± SD	12.7 ± 3.8	14.1 ± 2.9	0.14
Liver function tests				
	Bilirubin (Mean ± SD)	41.6 ± 8.7	44.5 ± 10.2	0.28
	ALP (Mean ± SD)	204.8 ± 30.2	194.8 ± 26.4	0.21
ERCP				0.48
	Done	22	20	
	Not done	4	6	

*BMI* body mass index, *ASA* American Society of Anesthesiologists, *ALP* alkaline phosphatase, *E* level of injury according to Strasberg classification, *CBD* common bile duct, *ERCP* endoscopic retrograde cholangiopancreatography

### Perioperative data among the study population

Patients who underwent RYHJ-C had comparable operative time (219.9 ± 34.2 vs 235.2 ± 21.4 (minutes); *P* <  = 0.059) and blood loss (312.5 ± 36.1vs 297.3 ± 18.9; *P* = 0.061) to those underwent RYHJ-GA. Ductoplasty was performed in fifteen cases; all of them underwent single anastomosis. External stenting was done in 2 cases whereas internal stenting in one case. Ileus, bile leakage, and wound infection occurred more in patients of the RYHJ-GA group than the RYHJ-C group, despite not reaching statistical significance (Table 2). No mortalities occurred in the first 30 postoperative days.

**Table 2:** Operative early postoperative data of the patients

		RYHJ-C (*n* = 26)	RYHJ-GA (*n* = 26)	*P* value
Operative time (minutes)	Mean ± SD	219.9 ± 34.2	235.2 ± 21.4	0.059
Blood loss (cc)	Mean ± SD	312.5 ± 36.1	297.3 ± 18.9	0.061
Ductoplasty		7	8	0.760
Stenting	Internal	0	1	0.552
	External	1	1	
Wound complications		2	4	0.39
Biliary leakage		4	5	0.71
Gastric leakage		–	0	NA
Ileus		4	7	0.31
Pulmonary complications		1	1	1.00
30-day mortality		0	0	NA
Hospital stay (days)	Mean ± SD	5.4 ± 1.3	6.1 ± 1.7	0.10

### Long-term surgical outcomes

Dyspepsia occurred in one patient of RYHJ-C and in two patients of RYHJ-GA that responded well to a short PPI regimen while incisional hernia occurred in 2 patients with RYHJ-C and one patient with RYHJ-GA.

In the RYHJ-C group, three cases had definite HJAS presented with attacks of cholangitis, persistent elevation of ALP and mild elevation of bilirubin (Table 3). Diagnosis was confirmed with MRCP. One case had intrahepatic stones. Two of the three cases underwent revisional surgery in the form of redo-hepatecojejunostmy with uneventful follow-up. The third patient refused surgery and was managed with medical treatment. Ursodexycolic acid (900 mg/day) was prescribed which alleviated the cholangitis and normalized the bilirubin level [29]. However, ALP was not normalized.

**Table 3:** Follow-up data of patients

	RYHJ-C (*n* = 26)	RYHJ-GA (*n *= 26)	*p*-value
Cholangitis	3	2	0.64
Stricture	3	4	0.68
Hepatolithiasis	1	2	0.55
Revisional surgery	2	1	0.55
Mean Follow up time (months)	29.9 ± 3.9	32.1 ± 4.2	0.056

**Fig. 3:**
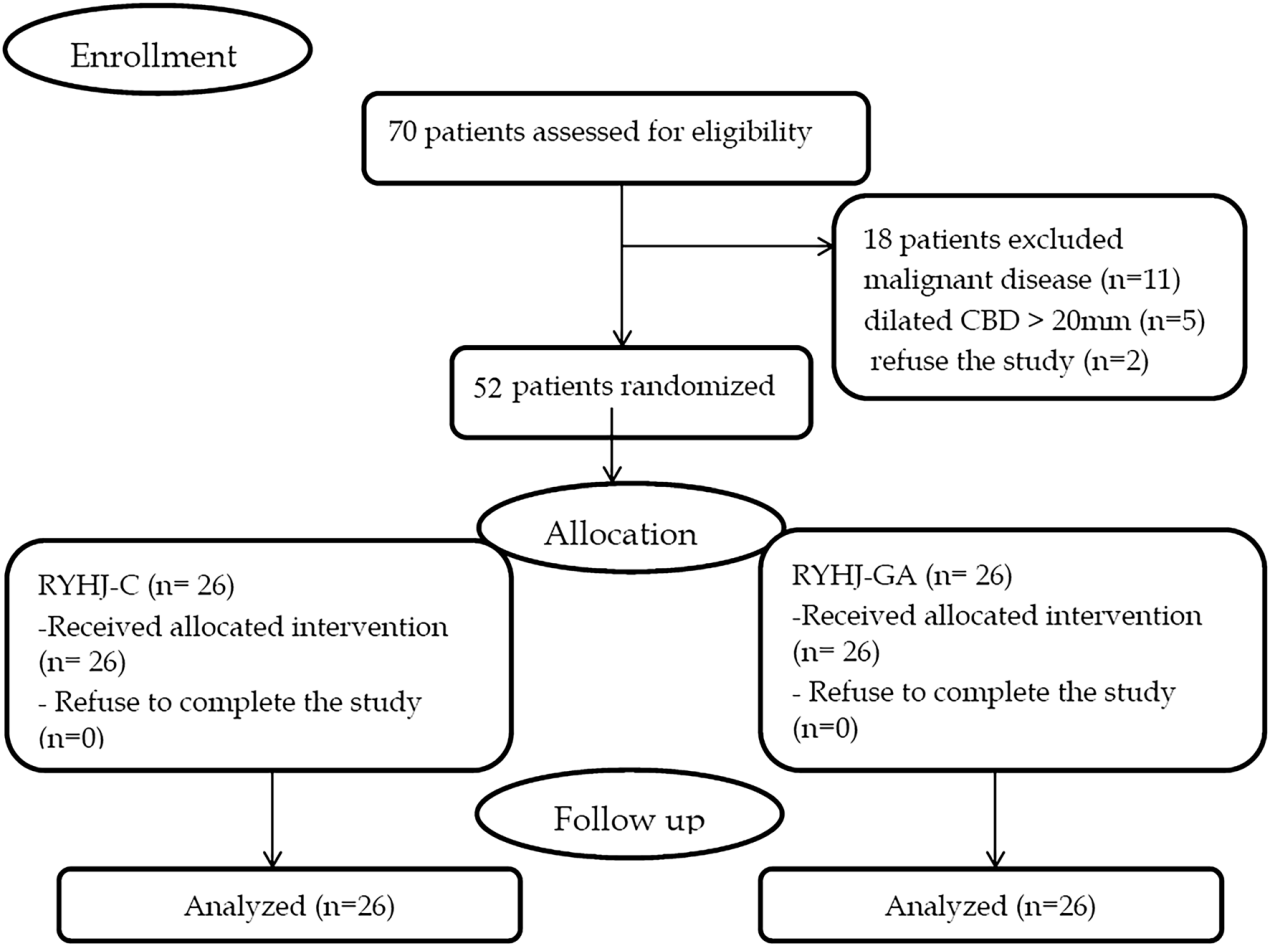
Consort flow diagram

In the RYHJ-GA group, three cases of 26 cases did not undergo the planned postoperative endoscopy due to patient refusal. One of them returned with biliary pain due to HJAS. Endoscopic access to the HJ was successful but the cannulation failed due to too tight stricture with subsequent failure of anastomotic stricture dilatation. Revisional surgery, in the form of excision of stricture and re-anastomosis leaving both GJ and entero-enterostomy intact, was done. Strict follow-up of this patient was done with prophylactic endoscopic dilatation (Fig. 3). Follow-up liver function tests were normal. The other two cases showed normal liver function test throughout the follow-up period.

**Fig. 4:**
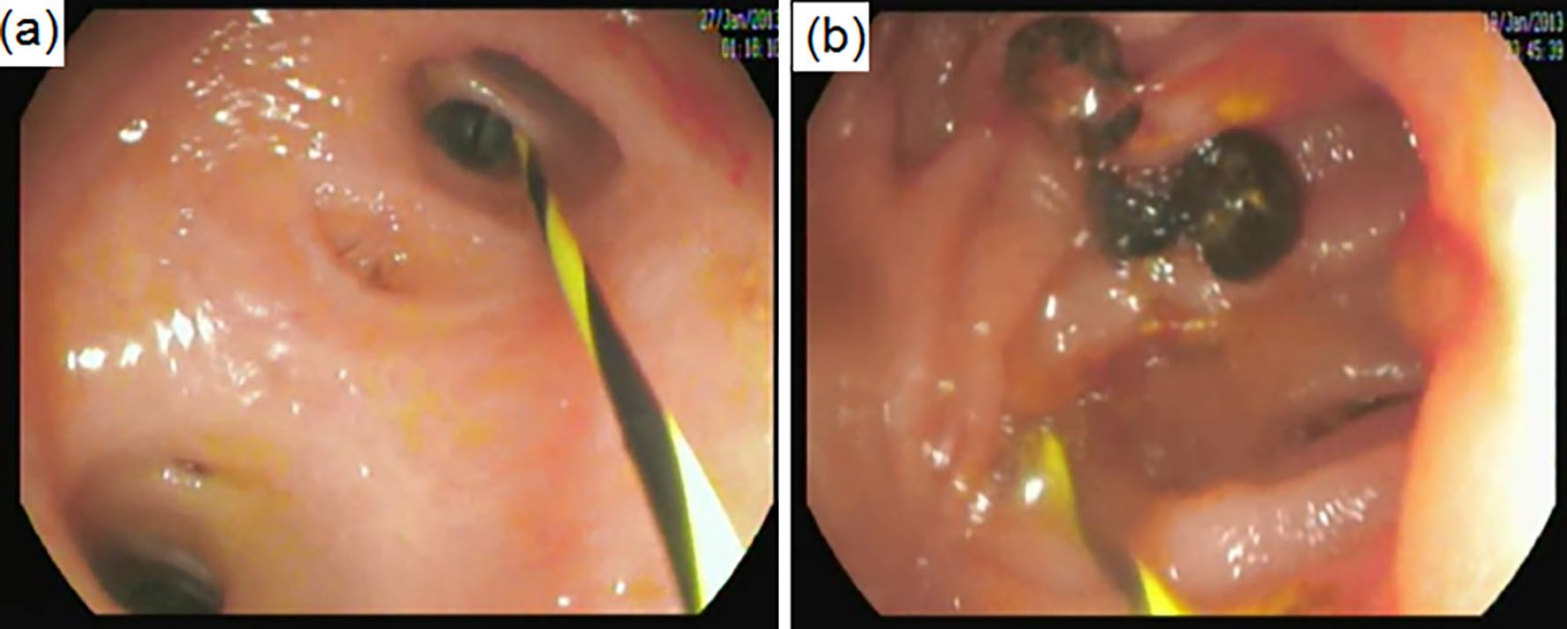
Endoscopic management of hepaticojejunostomy. **a** Cannulation of the hepatic duct in a patient who underwent hepaticojejunostomy and ductoplasty. **b** Balloon sweeping of intrahepatic stones

**Fig. 5:**
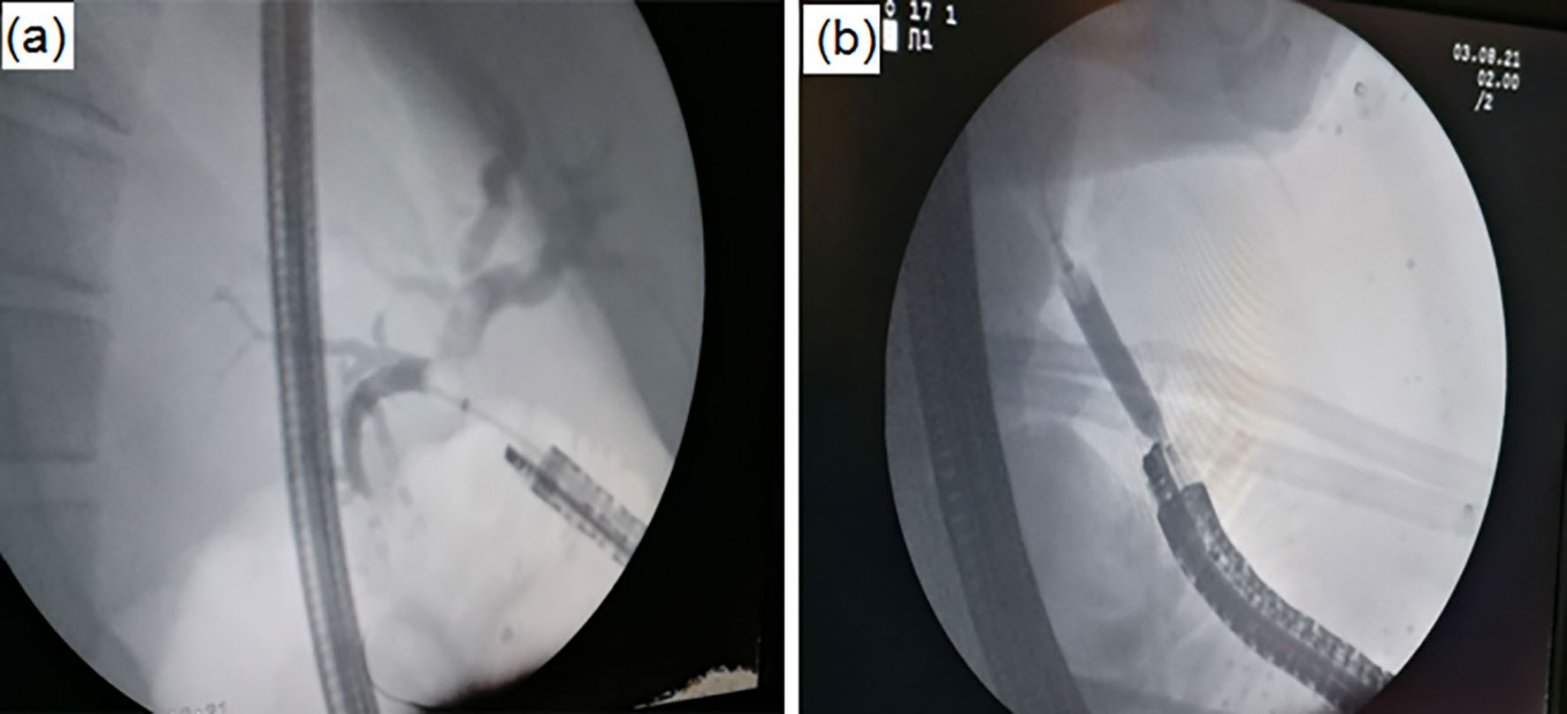
Cholangiogram via hepaticojejunostomy. **a** Dilated biliary channels with an anastomotic stricture. **b** Balloon dilatation of stricture

Of the remaining 23 cases, 20 cases underwent a single endoscopy session and 3 cases needed more than one endoscopy session due to significantly elevated ALP (one asymptomatic case needed another session), and cholangitis (2 cases, one of them needed another 2 sessions), (Table 4). All three cases were diagnosed by endoscopy to have HJAS and had successful endoscopic dilatation and balloon sweeping of biliary mud (one case) or stones (2 cases), without stenting (Figs. 4, 5). The follow-up liver function tests of all 3 cases returned to normal after successful endoscopic management.

**Table 4:** Endoscopic findings of RYHJ-GA cases

Number of patients underwent endoscopic assessment	24/26
Number of endoscopic sessions for every patient 1 session 2 sessions 3 sessions Total	21 2 1 28
Successful endoscopic cannulation of HJ	26/28 [93%] (1 case cannot see HJ; 1 case failed cannulation due to tight stricture)
Endoscopic evidence of gastritis	0/28
Bile in stomach Scale 0 Scale 1 Scale 2	6/28 [21.5%] 15/28 [53.5%] 7/28 [25%]
Time to reach HJ (mean ± SD)	12.5 ± 2.3
Stricture	4/26 [15.4%]
Successful dilatation of stricture cases	3/4 [75%]
Stones retrieval	2

## Discussion

Roux-en-Y hepaticojejunostomy is considered the procedure of choice for biliary reconstruction. However, HJAS remains a frequent serious long-term complication even in hepatobiliary centers [2–5, 7].

Revision of hepaticojejunostomy, performed to manage HJAS, is a technically challenging procedure that usually necessitates a high anastomosis in a hilar and intrahepatic board. This carries a morbidity of approximately 25% and a mortality of about 2%. In addition, a success rate of surgical repair decreases with each attempt for surgical intervention [5, 6, 16]. Percutaneous transhepatic balloon dilation for HJAS is an alternative option to redo-hepaticojejunostomy but has major limitations. These include the need for external biliary drainage which restricts the patient’s activity, high failure to gain access to a non-dilated biliary system, the need for multiple punctures and serial dilations, higher complication rates in patients with portal hypertension and/or coagulopathy, and the limited availability of high expertise in many centers [8–10]. Endoscopic management (via either balloon dilatation or stenting of the stricture) using a single- or double-balloon enteroscopy is a safe, highly effective option in patients with HJAS to avoid the need for revisional surgery [11–15]. However, endoscopic access to the HJ anastomotic site is difficult due to the lengthy pathway in Roux-en-Y reconstruction.

Many modifications of hepaticojejunostomy with the provision of an "access loop" were developed to shorten the distance traveled by the endoscope in the jejunal loop to reach the HJ anastomotic site. The earliest description of these modifications involved the fashioning of cutaneous stoma in the Roux limb of hepaticojejunostomy (hepaticocutaneousjejunostomy). This technique was abandoned due to excessive bile loss and unpleasant side effects of stoma [30–32]. Subparietal, subfascial or subcutaneous placement of the closed end of the afferent jejunal loop resulted in the difficulty of transjejunal endoscopy with the need for fluoroscopic assistance. Also, opening and closing the skin and jejunal loop were needed after each endoscopic procedure with the risk of wound infection or jejunal fistula [33–36]. Duodenal access loop was described by Stiegmann et al. [37]. Among seven patients in his report, successful endoscopic access to the biliary enteric anastomosis occurred in only three patients. In addition, his technique has a risk of ascending cholangitis [38].

Modified hepaticojejunostomy with gastric access loop was first innovated by Sitaram et al. in 1998 [21]. However, his technique did not gain popularity in most hepatobiliary centers and until recently, only a few publications studied the technique. This is probably due to four reasons: (1) the fear of biliary gastritis from the presence of bile in the stomach, (2) the risk of cholangitis from food particles entering the access loop, (3) Adding another anastomosis to the procedure with the risk of gastric fistula, and finally (4) the therapeutic benefit of endoscopic access to HJ anastomosis is questionable.

In our study, only two patients in RYHJ-GA reported dyspeptic symptoms shortly after the procedure which were managed effectively with PPI. Neither of them nor other patients who underwent endoscopic evaluation were found to have endoscopic evidence of gastritis. These results are in agreement with previous studies [17, 18, 20, 21]. Also, there is no case of GJ stomal ulcer, a complication reported in one patient of the sitaram et al. series [21]. On the other hand, most of the patients showed the presence of bile in the stomach. We believe that the nature of pure bile content in the stomach of our patients is totally different from that of the duodenogastric reflux which contains a mixture of bile and pancreatic enzymes. The presence of activated pancreatic enzymes is responsible for the injurious effects of reflux biliary gastritis and esophagitis [39, 40]. Moreover, the amount of bile in the stomach was significantly small in comparison to the copious amount of bile in the access loop (which was shown once the endoscope traversed the GJ). This may be explained by the strong gastric muscular layer which contracts during digestion closing the opening of GJ. Also, the bile flow to the stomach is upstream against the peristalsis of the jejunal loop.

The hypothesis that RYHJ-GA has a risk of cholangitis due to the entering of stomach contents into the access loop contradicts the results of our study and previous ones [17, 18, 20, 21]. No patient without stricture was reported to have cholangitic attacks. It is thought that during gastric emptying, the contracting gastric musculosa directs food to the patent-dependent pyloric orifice rather than the relatively closed GJ.

Adding another anastomosis to the RYHJ procedure has two potential disadvantages: the risk of gastric fistula and increased operative time. In our study, there is no case of gastric fistula. This is consistent with the previous studies [17, 18, 20, 21]. We suppose that the risk of gastric fistula is similar to the risk of intestinal fistula from enteroenterostomy, which is extremely rare in elective settings. Interestingly, operative time was comparable between RYHJ-C and RYHJ-GA groups (*P* = 0.059). This may be explained by the high variation in the difficulty of adhesiolysis and hilar dissection among cases which greatly affects the operative time rather than adding a GJ anastomosis to the standard procedure. In addition, early postoperative complications and length of hospital stay were comparable between both groups (*P* = 0.1).

Post-operative biliary leakage in the present study was 17.3% which is in the upper range reported in the literature [2, 41, 42], but insignificantly different in both groups (*P* = 0.71). Probably, the demanding anastomoses with narrow ducts or at a high level of hilar board performed in most cases of our series can justify these results. All cases of bile leak stopped spontaneously without intervention.

The main advantage of RYHJ-GA is the feasible endoscopic accessibility to HJ. The endoscopic access in RYHJ-GA is characterized by being via a natural orifice. This is in contrast to RYHJ with percutaneous biliary access including subcutaneous, subfacial and subparietal access loop reconstructions, in which endoscopic entry needs opening of the skin and jejunal loop with each endoscopic intervention with the risk of wound infection or jejunal fistula [33–36]. Although the reported technical success is high (ranges from 87.5% upto 100%), the clinical success (71.4%-75%) is not guaranteed [35, 43, 44]. In this study, endoscopy into the access loop via GJ was successful in all 24 patients in whom it was attempted. The endoscopic maneuver was easy with an end-view gastroscope, and we did not face any obstacle from GJ stricture that was reported in three out of eleven patients in the Selvakumar et al. series [20].

Three cases of RYHJ- GA needed more than one endoscopy session due to evidence of HJAS. It should be noted that not all RYHJ- GA patients underwent the first endoscopy at the planned time (3 months postoperative) due to the COVID-19 pandemic waves. Interestingly, two of these 3 cases underwent their first endoscopy at 5 and 7 months. Fortunately, all 3 cases had successful endoscopic dilation and balloon sweeping of biliary mud (one case) or stones (2 cases), without stenting. Although stenting after dilatation of biliary stricture remains a controversial issue [45, 46], we do not prefer stenting as it acts as a foreign body in the biliary system, leads to repeated episodes of cholangitis and encourages the formation of intrahepatic stones. The follow-up liver function tests of all 3 cases returned to normal after successful endoscopic management.

The incidence of HJAS was comparable between the two groups (*P* = 0.68). However, the follow-up period in this study is relatively short and we need a longer follow-up period for an accurate comparison of HJAS incidence between both techniques. Many studies reported that most HJAS develop within 5 years, and 90% within 7–10 years [5, 6]. Longer follow-up of our patients in the RYHJ-GA group who underwent endoscopic dilatation would clarify the benefit, if any, of this dilatation on the avoidance of future stricture formation. Moreover, those who may develop HJAS have the potential benefit of endoscopic management of their condition without the need for revisional surgery.

To our knowledge, this study is the first controlled comparative study that constitutes the largest number of patients who had hepaticojejunostomy with gastric access loop and had an endoscopic evaluation of the HJ anastomotic site (Table 5).

**Table 5:** Data of case series on hepaticojejunostomy with gastric access loop

	Total number of cases	Number of cases followed up	Follow-up period (months)	HJ stricture	Biliary gastritis	Success of Endoscopic access to HJ	Therapeutic use of gastric access loop
Sitaram et al. (1998)	10	7	3–24	1	0	4/5	None
Perakath et al. (2003)^a^	8	NA	NA	2	NA	2/2	Stricture dilation (2)
Selvakumar et al. (2008)	13	11	20–81 (Mean: 51)	1	0	8/11 GJ stricture (3)	None
Jayasundara et al. (2010)	27	25	6–61 (Mean 35.4)	3	5 cases of dyspepsia without endoscopic evidence	3/3	Stricture dilation (1) Stricture dilation and stenting (1) Balloon sweeping (1)
Hamad and El-Amin (2012)^b^	10	10	7–35 (Mean: 23)	1	0	10/10	Stricture dilation and stenting (1)
Current study	26	26	32.1 ± 4.2	4	2 cases of dyspepsia without endoscopic evidence	26/28	Stricture dilation and balloon sweeping (3)

*NA*-specific data is not available, *HJ* hepaticojejunostomy, **a** the original study discusses the need for portosystemic shunting before hepaticojejunostomy in patients with post-cholecystectomy benign biliary stricture and portal hypertension and data on follow up of gastric access loop patients was not available. **b** The original study discussed three different types of bilio-entero-gastrostomy (BEG) and data was extracted from BEG type III patients that had biliary reconstruction similar to other studies

Although our experience in this study is limited to 26 cases of RYHJ-GA, the simplicity and safety of the technique and the easy ability to access the HJ anastomotic site for balloon dilatation have convinced us that the procedure should be considered for patients in whom HJAS is anticipated such as patients with intra-abdominal abscess or bile collection, external biliary fistula, proximal biliary stricture, non-dilated biliary system, and prior attempts of repair.

## Conclusion

Modified Roux-en-Y hepaticojejunostomy with gastric access loop is comparable to the classic hepaticojejunostomy regarding complications. However, gastric access enables easy endoscopic access for the management of future HJAS. This modification should be considered in patients with a high risk of HJAS stricture during long-term follow-up. Randomized trials with a larger number of patients and longer follow-ups are needed to confirm these results.
